# Enhancing the Biological Relevance of Machine Learning Classifiers for Reverse Vaccinology

**DOI:** 10.3390/ijms18020312

**Published:** 2017-02-01

**Authors:** Ashley I. Heinson, Yawwani Gunawardana, Bastiaan Moesker, Carmen C. Denman Hume, Elena Vataga, Yper Hall, Elena Stylianou, Helen McShane, Ann Williams, Mahesan Niranjan, Christopher H. Woelk

**Affiliations:** 1 Faculty of Medicine, University of Southampton, Southampton SO17 1BJ, UK; a.heinson@soton.ac.uk (A.I.H.); y.p.gunawardana@soton.ac.uk (Y.G.); bastiaanmoesker@gmail.com (B.M.); 2 London School of Hygiene and Tropical Medicine (LSHTM), Department of Pathogen Molecular BiologyLondon WC1E 7HT, UK; carmen.denman@gmail.com; 3 iSolutions, University of Southampton, Southampton SO17 1BJ, UK; e.vataga@soton.ac.uk; 4 Public Health England, National Infection Service, Porton Down Salisbury, SP4 0JG, UK; yper.hall@phe.gov.uk (Y.H.); ann.rawkins@phe.gov.uk (A.W.); 5 The Jenner Institute, University of Oxford, Oxford OX3 7DQ, UK; elena.stylianou@ndm.ox.ac.uk (E.S.); helen.mcshane@ndm.ox.ac.uk (H.M.); 6 Department of Electronics and Computer Science, University of Southampton, Southampton SO17 1BJ, UK; mn@ecs.soton.ac.uk

**Keywords:** reverse vaccinology, machine learning, support vector machine, bacterial protective antigen, bacterial pathogen

## Abstract

Reverse vaccinology (RV) is a bioinformatics approach that can predict antigens with protective potential from the protein coding genomes of bacterial pathogens for subunit vaccine design. RV has become firmly established following the development of the BEXSERO^®^ vaccine against *Neisseria meningitidis* serogroup B. RV studies have begun to incorporate machine learning (ML) techniques to distinguish bacterial protective antigens (BPAs) from non-BPAs. This research contributes significantly to the RV field by using permutation analysis to demonstrate that a signal for protective antigens can be curated from published data. Furthermore, the effects of the following on an ML approach to RV were also assessed: nested cross-validation, balancing selection of non-BPAs for subcellular localization, increasing the training data, and incorporating greater numbers of protein annotation tools for feature generation. These enhancements yielded a support vector machine (SVM) classifier that could discriminate BPAs (*n* = 200) from non-BPAs (*n* = 200) with an area under the curve (AUC) of 0.787. In addition, hierarchical clustering of BPAs revealed that intracellular BPAs clustered separately from extracellular BPAs. However, no immediate benefit was derived when training SVM classifiers on data sets exclusively containing intra- or extracellular BPAs. In conclusion, this work demonstrates that ML classifiers have great utility in RV approaches and will lead to new subunit vaccines in the future.

## 1. Introduction

Reverse vaccinology (RV) is a form of vaccine research that uses bioinformatics approaches to identify putative vaccine candidates in the protein coding genomes of bacteria (i.e., proteomes). The most successful RV study to date was by Pizza and colleagues [1] and led to a subunit vaccine against *Neisseria meningitidis* (*N. meningitidis*) serogroup B [2]. Initially, open reading frames in the genome of *N. meningitidis* serogroup B were identified and bioinformatics programs (psortB [3], ProDom [4], and Blocks databases [5]) used to predict the subcellular localization for every protein in the proteome. Extracellular predicted proteins were then cloned and expressed as recombinant proteins, purified and had their predicted surface expression confirmed through techniques such as enzyme-linked imunosorbent assay (ELISA) and fluorescence activated cell-sorting (FACS). Proteins with confirmed surface expression were then used to generate antibodies in the serum of immunized mice and the bactericidal activity of this serum was assessed. Examples that met these criteria were then screened for conservation across multiple MenB strains and their suitability for manufacturing in bulk was assessed [6]. In the final subunit vaccine, BEXSERO^®^, three proteins were selected for incorporation, (i.e., Factor H binding protein, Neisserial adhesion A, and Niesseria heparin binding antigen) along with a detergent extracted outer membrane vesicle (DOMV). The BEXSERO^®^ vaccine is now licensed in over 35 countries and has already had an impact on the mortality and morbidity associated with *N meningitidis* serogroup B [7].

The RV approach of Pizza et al. [1] may be classified as a “filtering” approach, i.e., the organism’s proteome is passed through a series of filters until a subset of proteins are identified that represent potential vaccine candidates. Several utilities have been developed to implement filtering approaches to RV, for example Violin [8], Jenner Predict [9], and Ivax [10]. Drawbacks of filtering methods include the necessity of assessing large numbers of candidates in the laboratory and potential candidates with predicted subcellular localization other than extracellular (e.g., cytoplasmic) are discarded [11]. The latter is a significant limitation since proteins predicted to be cytoplasmic or of unknown localizations have been shown to confer significant levels of protection in animal models [12,13,14,15,16,17,18,19]. Machine learning (ML) approaches to RV circumvent these problems since they do not discard such proteins but are able to successfully model the entire proteome of a bacterial species and rank predicted antigens for their likelihood of being a vaccine candidate [20,21].

The first ML study in the field of RV was published by Doytchinova and Flower [21], in which a training dataset was generated of 100 known antigens through a literature curation that defined a known antigen as a protein (or part of a protein) that, “has been shown to induce a protective response in an appropriate animal model after immunization”. A negative training dataset was constructed by randomly sampling 100 proteins or non-antigens from the same bacterial species that corresponded to each known antigen in the positive training dataset. The proteins in this training dataset were annotated with auto cross-covariance (ACC) transformations, which reflect hydrophobicity, molecular size, and polarity. The annotated proteins were used to train a classifier based on discriminant analysis by partial least squares (DA-PLS), which was able to achieve an accuracy of 82% when distinguishing non-antigens from known antigens. In an extension to Doytchinova and Flower’s [21] work, our initial RV study [20] focused exclusively on bacterial protective antigens (BPAs) defined as, “a whole protein that led to significant protection (*p* < 0.05) in an animal model (i.e., bacterial load reduction or survival assay) following immunization and subsequent challenge with the bacterial pathogen”. Focusing on bacterial proteins the size of the training data (136 BPAs and 136 non-BPAs) was increased and then annotated with biologically-relevant protein annotation tools (e.g., PSORTb [3], LipoP [22], and Bepipred [23]) for the training of support vector machine (SVM) classifiers. This work showed that higher accuracies were obtained when using SVMs (i.e., 92%) when separating BPAs and non-BPAs in the training data and when recalling known antigens in the background of entire bacterial proteomes [20].

Building on our previous work in the field of ML applied to RV, this current study implemented a nested approach to cross-validation, removed an artificial bias associated with the selection of non-BPAs for the negative training data, increased the size of the training data by approximately a third, and incorporated new protein annotation tools to model different aspects of immunogenicity (e.g., T-cell epitope prediction and Adhesin prediction [24]). The resulting SVM classifier was used to demonstrate that a significant signal for protection could be captured through the literature curation of BPAs as assessed through comparisons to randomly-permutated data.

## 2. Results

### 2.1. Permutation Analysis Reveals a Strong Protective Signal for BPAs Curated from the Literature

To enhance the biological relevance of ML classifiers for RV, several modifications were made to our previous approach [20]. These included adopting a nested (N) cross-validation approach, balancing (B) the negative training data for subcellular localization, increasing the size of the positive training dataset to 200 BPAs, and increasing the number of protein annotation tools by 15 to derive a total of 525 annotation features (AF) (full list Table S1). This resulted in a training data set with the designation BPAD200+N+B+AF consisting of 200 BPAs and 200 non-BPAs annotated with 525 features. Non-BPAs in the negative training data were selected by randomly sampling proteins from the same bacterial proteome for the relevant BPA pair while ensuring the same subcellular localization.

**Figure 1:**
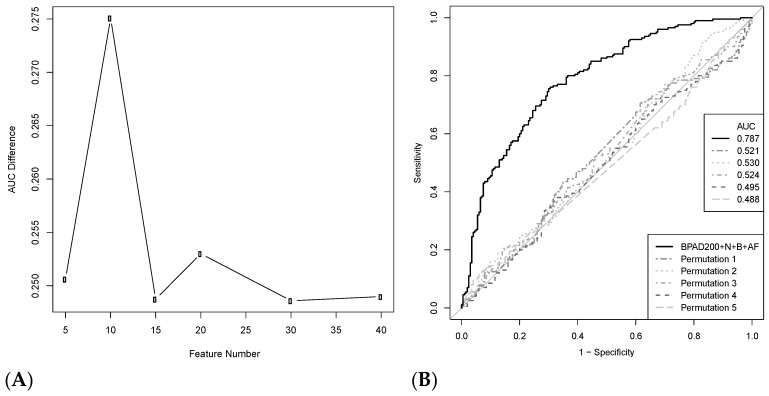
(**A**) Plot of the difference in area under the curve (AUC) between the support vector machine (SVM) classifier BPAD200+N+B+AF versus randomly permutated data with increasing feature numbers. SVM classifiers were trained to discriminate bacterial protective antigens (BPAs) and non-BPAs in BPAD200+N+B+AF and receiver operator characteristic (ROC) curves generated from a nested leave tenth out cross-validation approach for different numbers of features selected by greedy backward feature elimination. Five iterations were performed to assess the random breakage of ties during greedy backward feature elimination and AUC was averaged across iterations for each feature set. This analysis was then repeated for five datasets where the BPA and non-BPA labels were randomly permutated and average AUC calculated across randomly permutated data sets for each feature set; (**B**) ROC curves for the average of the five iterations of the 10 feature SVM classifier derived from BPAD200+N+B+AF (black solid line) and from each of the five randomly-permutated datasets (dotted grey lines).

Initially, the BPAD200+N+B+AF dataset was used to determine if a signal for protective efficacy had been captured when curating BPAs from the literature record. SVM classifiers were trained in a nested leave tenth out cross-validation (LTOCV) approach to discriminate BPAs from non-BPAs in BPAD200+N+B+AF and in five additional data sets where the labels (i.e., BPA or non-BPA) were randomly permutated (permutation testing) and, thus, contained no biological information. Greedy backward feature elimination was used to select different numbers of features for SVM classifiers. For each feature set, the difference in the AUC calculated from receiver operator characteristic (ROC) curves between BPAD200+N+B+AF and the randomly permutated data was calculated (Figure 1A). An SVM classifier with the 10 most informative features led to a significant separation in AUC (average *p*-value 1.13 × 10^−12^, DeLong test [25]) between BPAD200+N+B+AF and the randomly permutated data (Figure 1B). These results clearly demonstrated that the literature curation of BPAs captured a strong protective signal and that 10 features was the optimal number for discriminating BPAs from non-BPAs. Therefore, SVM classifiers containing 10 features were used to evaluate the changes in classification accuracies of each of the modifications to our RV approach.

### 2.2. A Nested Approach Has a Significant Impact on the Ability of SVMs to Classify BPAs

**Figure 2:**
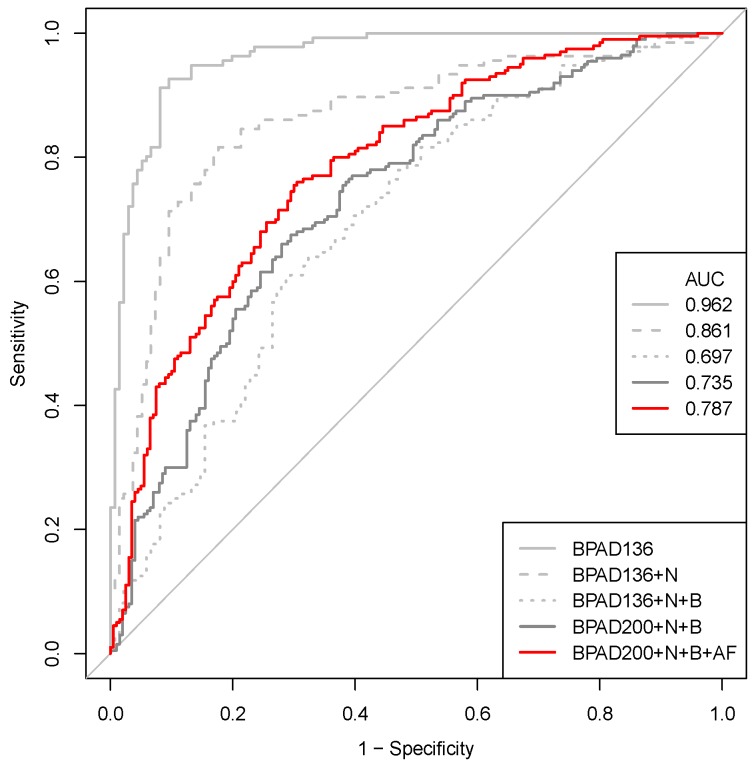
ROC curves were generated from SVM classifiers utilizing 10 features selected by greedy backward feature elimination in a LTOCV approach. Averages were plotted across five iterations of SVM classifiers implemented to randomly break ties resulting from the greedy backward feature elimination procedure. The benchmark to assess these modifications was a non-nested, non-balanced training data set of 136 BPAs and 136 non-BPAs annotated with 122 features from 19 protein annotation tools (BPAD136) [20]. Subsequent modifications were added in a stepwise fashion and included: a nested cross-validation approach (BPAD136+N), balanced selection of non-BPAs for predicted subcellular localization (BPAD136+N+B), increased size of training data (BPAD200+N+B), and additional features (525 total) derived from an increased number of protein annotation tools (BPAD200+N+B+AF).

Having demonstrated that the SVM classifier BPAD200+N+B+AF trained on datasets curated from the literature had captured a biological signal reflective of protective antigens, the multiple modifications made to our RV approach were assessed in a stepwise manner. The starting point for this assessment was the BPAD136 classifier from our previous work [20], consisting of 136 BPAs and 136 non-BPAs. The following modifications to BPAD136 were then assessed in turn: nested cross-validation (BPAD136+N), balancing non-antigen selection for subcellular localization (BPAD136+N+B), increasing the size of training data, (BPAD200+N+B) and, finally, incorporating additional features (BPAD200+N+B+AF). Our previous work [20] had not implemented a truly nested cross-validated approach and it was hypothesized that previous SVM classifiers may have overfit the data. This was indeed the case, as reflected by a significant reduction in AUC (*p*-value = 9.69 × 10^−5^, DeLong test [25]) when migrating from an overfit (BPAD136, AUC = 0.962) to a nested (BPAD136+N, AUC = 0.861) approach (Figure 2). Therefore, the implementation of a nested approach to cross-validation is recommended for RV studies and enables a better estimation of the performance of SVM classifiers.

### 2.3. Correcting a Bias in the Selection of Negative Training Data Impacts SVM Classification of BPAs

**Figure 3:**
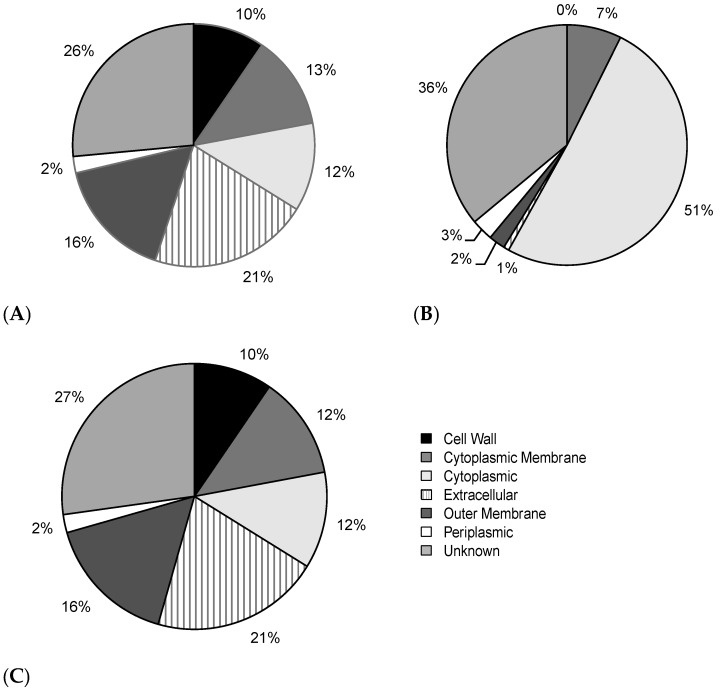
Pie charts showing subcellular localization as predicted by PSORTb [3] for the numbers of BPAs and non-BPAs in the following subsets of the BPAD136 dataset. (**A**) positive training data (i.e., 136 BPAs); (**B**) negative training data (i.e., 136 non-BPAs); and (**C**) negative training data balanced for subcellular localization (i.e., 136 non-BPAs).

The subcellular localizations predicted by PSORTb [3] were compared between the positive (BPAs) and negative (non-BPAs) training data for BPAD136. This demonstrated that the negative training data had a larger proportion of proteins predicted to be located in the cytoplasm (Figure 3A,B). This resulted from the random selection of non-BPAs for the negative training data. Specifically, for each BPA, a non-BPA was randomly selected from the proteome of the same bacterial species. Since the majority of proteins in any given bacterial proteome are predicted to be cytoplasmic, random sampling led to a disproportionate number of non-BPAs with this subcellular location in the negative training data. To correct this bias, a new negative training dataset was generated, where each non-BPA was selected to match not only the bacterial species of the corresponding BPA, but also its subcellular localization (Figure 3C). Removing this bias in subcellular localization decreased the ability of the SVM classifier to discriminate between BPAs and non-BPAs as reflected in a significant reduction in AUC (*p*-value of 3.44 × 10^−5^, DeLong test [25]) when comparing BPAD136+N to BPAD136+N+B (Figure 2). The performance of the SVM classifier is reduced because it can no longer utilize differences in subcellular localization to discriminate BPAs from non-BPAs. This is reflected by the removal of features related to bacterial subcellular localization (e.g., PSORTb and SignalP) from the top 10 features utilized by the classifier.

### 2.4. Increasing the Size of the Training Data Has a Positive Impact on the Ability of SVMs to Classify BPAs

A literature curation yielded 64 new BPAs that were significantly protective (*p* < 0.05) in an animal model following immunization and subsequent challenge with the bacterial pathogen. These BPAs were used to expand the positive training data set from 136 to 200 BPAs and paired with non-BPAs balanced for subcellular localization to form the training data set designated BPAD200+N+B. Increasing the size of the training data in this manner led to improvements in AUC (Figure 2, 0.697 to 0.735), although this change was not statistically significant.

### 2.5. Increasing the Number of Protein Annotation Tools Enhances the Ability of SVMs to Classify BPAs

The effect of incorporating new annotation features derived from protein annotation tools (Table S1) with biological relevance for immunogenicity (e.g., Spaan [26], MHCpan [27], GPS-MBA [28]) on the ability of SVM classifiers to distinguish BPAs from non-BPAs was examined. Compared to our original approach [20], an additional 15 protein annotation tools were assessed resulting in a training data set (BPAD200+N+B+AF) annotated with a total of 525 features. The incorporation of these additional features resulted in an SVM classifier with an increased AUC (0.787) when compared to BPAD200+N+B. Although this increase did not attain significance, it should be stressed that when the incorporation of additional BPAs and features were considered together (i.e., BPAD136+N+B to BPAD200+N+B+AF) there is a significant increase in AUC (*p* = 2.11 × 10^−2^, DeLong test [25]). Finally, the top 10 features selected by greedy backward feature elimination for the discrimination of BPAs and non-BPAs in the BPAD200+N+B+AF dataset contained new features primarily related to T-cell epitopes (Table 1).

### 2.6. Intracellular and Extracellular BPAs Utilize Different Features for Classification

**Figure 4:**
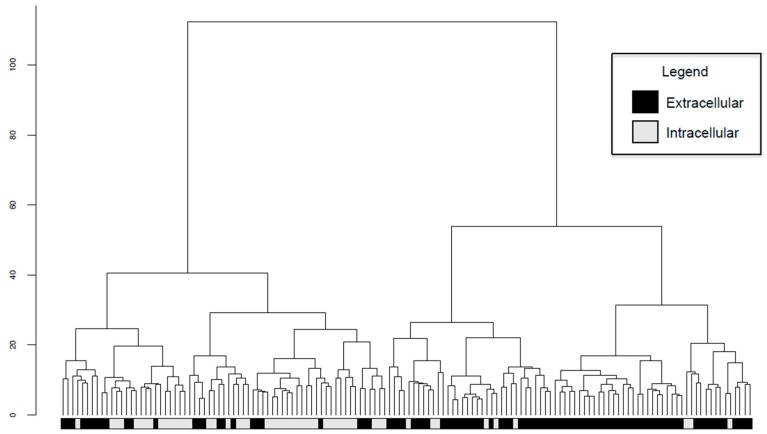
Hierarchical clustering of 142 BPAs from BPAD200+N+B+AF using all 525 annotation features, distances between BPAs were calculated using Euclidean metrics and then clustered using the Ward algorithm. White labels at the branch tips refer to BPAs with subcellular localization predicted by PSORTb [3] as intracellular (i.e., cytoplasm or cytoplasmic membrane) and black labels as extracellular BPAs (i.e., extracellular, periplasmic, outer membrane, cell wall).

An unsupervised approach was used to further explore the biological signals in the features derived from protein annotation tools used to annotate the 200 BPAs curated from the literature record. Hierarchical clustering of the 142 BPAs not predicted to have an unknown subcellular localization by psortB [3] using all 525 annotation features revealed two main groups that primarily corresponded to predicted subcellular localization, i.e., intra- or extracellular (Figure 4). This suggests that intracellular and extracellular BPAs may have fundamentally different biological properties. Therefore, it was hypothesized that greater accuracies would be achieved when SVM classifiers were trained separately on intra- and extracellular BPAs. To test this hypothesis, two training datasets were constructed from BPAD200: intracellular BPAD51 (iBPAD51) consisting of 51 BPAs and 51 non-BPAs with subcellular localization predicted as intracellular, and extracellular BPAD91 (eBPAD91) with 91 BPAs and 90 non-BPAs with subcellular localization predicted as extracellular. A matching non-BPA balanced for subcellular localization could not be found that met the inclusion criteria for ACF35754.1 (a BPA from *Salmonella enterica subsp. enterica serovar Paratyphi A*), detailed in the Methods (Section 4.1) and, thus, the negative training data was reduced by one.

**Figure 5:**
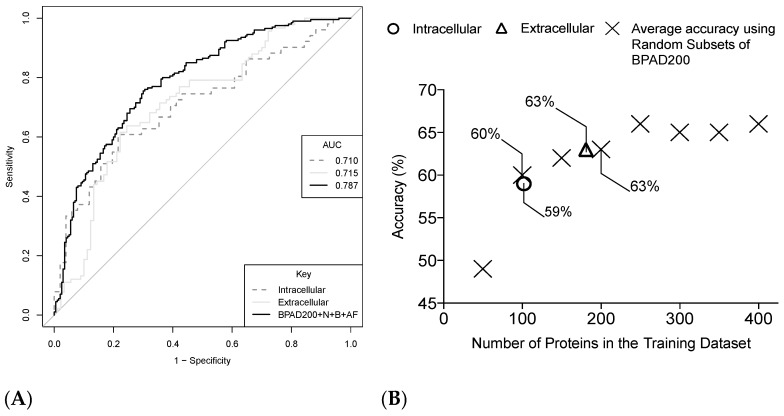
(**A**) ROC curves obtained from SVM classifiers trained to distinguish BPAs from non-BPAs in the following data sets: iBPAD51 (dotted line), eBPAD91 (solid grey line) and BPAD200+N+B+AF (black line). Curves were drawn by averaging results from five iterations of SVM classifiers consisting of 10 features selected by greedy backward feature elimination assessed in a LTOCV approach; (**B**) Plot showing the average percentage accuracy (five iterations) of SVM classifiers of 10 features trained on different sized subsets of BPAD200+N+B+AF for comparison to SVM classifiers derived from iBPAD51 and eBPAD91.

Contrary to expected findings, SVM classifiers trained on iBPAD51 and eBPAD91 had lower AUC values compared to those trained on BPAD200 (Figure 5A). However, SVM classifiers had been trained on datasets of different sizes and this might explain the superior performance of BPAD200, which was the largest dataset. Therefore, SVM classifiers were trained on randomly selected subsets of BPAD200 of decreasing size (Figure 5B). This facilitated a better comparison of intra- (trained on iBPAD51) or extracellular (trained on eBPAD91) SVM classifiers, versus those trained using BPAs and non-BPAs from all subcellular localizations (BPAD200). However, the accuracies derived from iBPAD51 and eBPAD91 were similar to data sets of similar size consisting of BPAs from all subcellular localizations (Figure 5B). This suggests there is no immediate benefit to training separate SVM classifiers to recognize intra- or extracellular BPAs.

Finally, to fully explore differences between SVM classifiers trained on intra- (iBPAD51) and extracellular (eBPAD91) BPAs, the top 10 features selected by greedy backward feature elimination for each classifier were interrogated (Table 2). The SVM classifier trained on eBPAD91 (extracellular classifier) utilized features derived from protein annotation tools that were not utilized by the classifier trained on iBPAD51 (intracellular classifier) and were related to the following diverse array of biological phenomena: an adhesin predictor SPAAN [26] (which describes if the protein adheres to the surface of cells), surface accessibility, and secondary structure predictor NetSurfP [29] (which predicts the relative likelihood of sections of each protein being exposed on the surface of a protein structure), lipoprotein prediction LipoP [22] (which predicts if a protein will interact with lipids), as well as a cleavage site predictor NetChop [30] (which predicts if a protein will be chopped by the human proteasome) (Table 2A). Features derived from protein annotation tools that were unique to the SVM classifier trained on iBPAD51 were largely related to immunogenicity and included: a B-cell epitope predictor [23] (predicts possible binding sites for B-cells), a T-cell epitope predictor [28] (predicts possible binding sites for T-cells) and a calpain cleavage predictor [31] (predicts a specific type of protein cleavage dependent on the presence of Ca^2+^) (Table 2B). In summary, SVM classifiers appear to utilize features from different protein annotation tools to discriminate intra- and extracellular BPAs from their respective non-BPAs. There may be benefit to deriving separate classifiers for both types of BPA in the future as the literature record expands allowing larger training data sets to be interrogated.

## 3. Discussion

An SVM classifier capable of discriminating BPAs from non-BPAs was evaluated in a fully-nested approach while examining the impact of the addition of new BPAs curated from the literature record and additional annotation features derived from new protein annotation tools. The major finding was that a signal of protective efficacy can be curated from published data in the form of BPAs defined in this study. This was evident from the significant drop in accuracy of SVM classifiers trained on randomly permutated data (Figure 1). To reiterate, a BPA was defined as a whole protein that led to significant protection (*p* < 0.05) in an animal model (i.e., bacterial load reduction or survival assay) following immunization and subsequent challenge with the bacterial pathogen.

The most biologically relevant classifier generated in this study was built upon the dataset designated BPAD200+N+B+AF (Figure 2). The top 10 features used by this classifier may be examined to determine which features are reflective of protective efficacy (Table 1). The main signal related to protective efficacy in these top 10 features was the prediction of T-cell epitopes (bioinformatics tools NetMHC [27] and MBAAgl7 [28]) with a greater number of epitopes associated with protection as expected. In addition, annotation related to general biological processes, such as threonine glycosylation, phosphorylation, and lipoproteins, are positively linked with protection. These processes have previously been implicated in controlling both the humoral and cellular immune responses [32,33,34,35,36]. For example, lipoproteins have been shown to increase the influence of major histocompatibility complex-II (MHC-II) activation on T-helper cells (Th cells) [33]. This is achieved by lipid rich microdomains co-localizing and increasing the MHC-II molecules concentration on the cell surface, resulting in more efficient Th cell activation while requiring less antigen [33]. In summary, although epitope prediction clearly has value in an RV approach, other tools predicting general protein annotation features should also be considered and continue to be incorporated in future enhancements.

Clustering BPAD200+N+B+AF revealed that BPAs grouped based on extracellular and intracellular predicted subcellular localization (Figure 4). Unexpectedly, separate Intracellular (trained on iBPAD51), Extracellular (trained on eBPAD91), and Combined (BPAD200+N+B+AF) classifiers achieved similar accuracies (Figure 5). However, it was theorized that intracellular and extracellular classifiers would be selecting different features in order to capture biological differences whilst making predictions of BPA or non-BPA. A logical hypothesis would be that extracellular classifiers utilize features related to B-cell epitopes since this antigen type is surface exposed and that intracellular classifiers require features related to T-cell epitopes since this antigen type is internalized. However, this was not exactly the case, whereby the intracellular classifier utilizes features from both B-cell and T-cell epitope predictors but the extracellular classifier does not utilize features from any epitope prediction tools (Table 2). This could be due to the difficulty in predicting conformational epitopes from amino acid sequences [37], it is estimated that 90% of B-cell epitope binding is conformational [38]. To model this information CBTOPE [39] (a conformational B-cell epitope predictor) was included in this study, however annotation features derived from CBTOPE were not present in the top ten annotation features used in classification for any of the classifiers trained in this study. It is possible that with more advanced techniques these conformational epitopes will be more accurately predicted from amino acid sequences and may become an important part of the classification for extracellular BPAs from non-BPAs. Instead the extracellular classifier uses annotation features derived from more general protein annotation tools (e.g., adhesin prediction, surface accessibility, and general cleavage site prediction). Although the utility of separate intra- and extracellular classifiers has not been demonstrated in this study it is clear that these classifier types are modelling different aspects of antigen biology and future studies will explore this as more data becomes available.

There are a number of limitations that may be affecting the ability of SVM classifiers to discriminate BPAs from non-BPAs. Firstly, it is possible that the random selection of non-BPAs for the negative training data may result in the mistaken addition of protective antigens (i.e., BPAs) that simply have not been tested and documented in the literature record. To negate this limitation, non-BPAs with homology to BPAs were discarded. Furthermore, permutation analysis demonstrated that the non-BPAs represent useful negative training data since there was clearly a discriminatory signal between BPAs and non-BPAs (Figure 1B). Another limitation is that the features on which the classifiers are trained are all generated from protein annotation tools. These tools are not 100% accurate and therefore some tools will introduce noise into the annotation used to train SVM classifiers. Future studies should continue to add new features from protein annotation tools as they are developed to asses if improvements to classifier accuracy can be achieved. Regardless, the annotation features derived from protein annotation tools currently available produce sufficient signal to significantly distinguish classifiers from randomly permutated data (Figure 1B). Finally, most antigens previously tested and confirmed in the literature are predicted to be of unknown subcellular localization (29%) or are extracellular (45.5%, eBPAD of BPAD200). This led to a small training dataset (iBPAD51) when building the intracellular classifier described in this study (51 BPAs and 51 non-BPAs). Incorporating more proteins in the training datasets may enable better description of differences in protection derived through intracellular or extracellular proteins.

It is envisaged that the application of ML approaches to RV will build upon the success of filtering approaches that lead to the BEXSERO^®^ vaccine. The SVM classifiers constructed in this study discriminate BPAs from non-BPAs and through comparisons to randomly permutated data clearly demonstrate that a signal for protective efficacy can be curated from the literature record. Future studies should concentrate on the expansion of the training data through the addition of further BPAs curated from the literature record and the incorporation of new protein annotation tools since these enhancements significantly increased the accuracy of SVM classifiers. This will increase the accuracy with which BPAs are predicted and reduce the number of laboratory assays that need to be performed in order to identify novel antigens with the required protective efficacy.

## 4. Methods

### 4.1. Training Data

A literature curation identified 64 new BPAs which were combined with 136 previously characterized BPAs [20] for a positive training dataset totaling 200 BPAs. A BPA is a bacterial protein that has led to significant protection (*p* < 0.05) in an animal model (i.e., bacterial load reduction or survival assay) following immunization and subsequent challenge with the bacterial pathogen. BPAs were only selected for inclusion if a consensus for this definition was met by two or more curators. Negative training data (non-BPA) was generated by randomly selecting a protein from the same bacterial species for each BPA. BLASTP was used to discard any non-BPAs that matched to known BPAs (i.e., >98% similarity) or non-BPAs already selected (*E*-value < 10 ×10^−3^) [20]. Similarity is a measure of the extent to which sequences are related and *E*-values are a representation of the same sequence occurring by chance, both are described fully in the NCBI BLAST help manual [40]. In addition, unless otherwise stated, non-BPAs were selected from the same predicted subcellular localization as their BPA partner. One BPA (ACF35754.1) did not have a predicted extracellular protein (same subcellular localization) that matched the BLASTP inclusion criteria and a protein with unknown subcellular localization was sampled instead for inclusion in the negative training dataset. In summary, a dataset consisting of 200 BPAs and 200 non-BPAs was constructed and referred to throughout this study as BPAD200.

### 4.2. Permutation Analysis

Permutation analysis was used to determine the optimal feature number for SVM classifiers. Labels (BPA and non-BPA) were randomly permutated five times, generating five datasets. Permutation analysis should destroy the antigenic signal and is fully described by Good [41]. The AUCs achieved when training SVM classifiers using greedy backward feature elimination on these datasets were averaged and compared to five iterations of greedy backward feature elimination of BPAD200+N+B+AF. This process was repeated with SVM classifiers trained with different numbers of features.

### 4.3. Data Annotation

A second literature curation identified new protein annotation tools to generate novel annotation features for training SVM classifiers. This study increased the number of protein annotation tools from 19 in our previous work [20] to 34, and the output from these tools was parsed to generate 525 annotation features. Annotation tools included in the previous study [20] had their outputs parsed in a standardized manner for all annotation tools. Protein annotation tools for eukaryotic proteins were initially included to maximize the data on which SVM classifiers were trained. Classifiers trained on datasets including additional annotation features not previously utilised in ML approaches to RV have the AF tag, and a full list of protein annotation tools and annotations features can be found in Table S1.

### 4.4. Machine Learning Classification

Annotation features were scaled individually between −1 and 1 before training SVM classifiers on BPAs and non-BPAs [42]. A table of scaled annotation features as submitted to the classification pipeline can be found as Table S2. All implementations of the SVM in this manuscript used a non-specific filtering step based on *F*-score [43,44] to reduce the number of annotation features to 200. These features then underwent greedy backward feature elimination [45] to remove the least informative feature each round until the desired number of features remained. Backward selection was used to enable the interaction of all features to be considered. When features have equal information content the algorithm randomly selects which feature to remove, SVM classifiers were trained in five separate iterations to assess the impact of randomly breaking such ties. A full description of SVM classifiers has been published by Noble [46].

To enable comparison to previous RV studies [20] SVMs with a radial bias function (RBF) kernel were used to construct classifiers, as implemented in the *libsvm* package [42] in Python following the *libsvm* user manual guidelines [47]. Unless otherwise indicated, classifiers were evaluated using a nested LTOCV model [48] to obtain SVM predicted probabilities for each protein within the training data of BPAs and non-BPAs. The first step in a fully nested approach was to split the data into 10 parts and isolate one of these tenths as the test dataset. Feature selection and parameter optimization were applied to the remaining training dataset only (i.e., the remaining 9/10 of the data). An SVM classifier was then built on only the training dataset and used to predict the class (BPA or non-BPA) of the test data in the one-tenth left out. This process was repeated a further nine times leaving the remaining tenths of the data out one at a time.

### 4.5. Statistics

ROC curves were used to evaluate the performance of SVM classifiers in this study, and generated by plotting the true positive rate (TPR, i.e., sensitivity) over the false positive rate (FPR, i.e., 1−specificity) [49]. Area under the curve (AUC) values were calculated from ROC curves and differences between AUC values was assessed with the DeLong [25] statistical test and *p* < 0.05 considered significant.

### 4.6. Hierarchical Clustering

The subcellular localization of BPAs from BPAD200+N+B+AF was predicted using the protein annotation tool PSORTb [3]. A BPA was labeled as extracellular if it was predicted to be localized close to the surface of the cell (i.e., cell wall, extracellular, outer membrane, or periplasmic). BPAs with a predicted subcellular localization of periplasmic were included in the extracellular group as these proteins clustered predominantly with the extracellular opposed to intracellular group. If a BPA was predicted to be localized to the cytoplasm or cytoplasmic membrane it was defined as intracellular. Intracellular and extracellular BPAs from BPAD200 were clustered on all annotation features using a Euclidean distance calculation and a Ward clustering metric using the *ClassDiscovery* [50], and *Dendextend* [51] packages in R [47].

## Figures and Tables

**Table 1:** The top 10 annotation features selected by greedy backward feature elimination for discrimination of BPAs from non-BPAs by the SVM classifier trained on the BPAD200+N+B+AF data set.

Rank	Feature	Name of Bioinformatics Tool	Protein Annotation Tool Type	Correlated with BPA or Non-BPA
1	LipoP_Signal_Avr_Length	LipoP	Lipoprotein	BPA
2	YinOYang-T-Count	YinOYang	Glycosylation	BPA
3	NetPhosK-S-Count	NetPhosK	Phosphorylation	BPA
4	LipoP_SPI_Avr_Length	LipoP	Lipoprotein	BPA
5	**NetMhcPan-B-AvgRank**	**NetMhc**	T-Cell Epitope predictor (MHC Class II)	BPA
6	TargetP-SecretFlag	TargetP	Subcellular Compartmentalisation—In Eukaryotic Cells	BPA
7	YinOYang-Average-Difference1_Length	YinOYang	Glycosylation	Non-BPA
8	**MBAAgl7_CorCount**	**GPS-MBA**	T-Cell Epitope predictor	BPA
9	**PickPocket-Average_score**	**PickPocket**	MHC Peptide Binding	Non-BPA
10	PropFurin-Count_Score	ProP	Cleavage Sites—In Eukaryotic Cells	BPA

Features in bold represent those derived from protein annotation tools that were added in this study compared to our previous approach [20]. For a full list of bioinformatics tools utilized in this study and the annotation features derived from them please see Table S1.

**Table 2:** The top 10 annotation features selected by greedy backward feature elimination utilized by SVM classifiers trained on (**A**) eBPAD 91 and (**B**) iBPAD51.

**(A)**			
**Rank**	**Feature**	**Protein Annotation Tool Type**	**Rank in Intracellular Classifier**
1	Pad-value	Adhesin	42
2	DictOGlyc_Ser_Average_Threshold_Length	Glycosylation	189
3	LipoP_SPI_AvrScore	Lipoprotein	NF
4	Netsurfp_RSA_Exposed_AverageDiff	Surface accesibility and secondary structure	NF
5	PoloPhosphorylation_CorAvg	Phosphorylation	NF
6	Net_Chop_CorCount	Predicts cleavage sites	NF
7	DictOGlyc-No_Score_Sites_Length	Glycosylation	NF
8	GPS_SUMO_Sumoylation_Average_Score	Small ubiquitin like modifiers (SUMOs) binding site prediction	9
9	ProtParam-PercIsoleucine	General Annotation	144
10	ProtParam-PercGlutamicAcid	General Annotation	NF
**(B)**			
**Rank**	**Feature**	**Protein Annotation Tool Type**	**Rank in Extracellular Classifier**
1	Bepipred-Count_Length	B-Cell Epitope	149
2	CCD_av_diff	Calpain Cleavage	NF
3	YinOYang-T-Average-Difference1_Length	Glycosylation	NF
4	ProtParam-GRAVY	General Annotation	35
5	NetOGlyc-T-Max-I	Glycosylation	196
6	YinOYang-T-Average_Length	Glycosylation	NF
7	ProtParam-PercAlanine	General Annotation	97
8	NetPhosK-Y-MaxScore	Phosphorylation	NF
9	GPS_SUMO_Sumoylation_Average_Score	Small Ubiquitin like modifiers (SUMOs) binding site predictor	8
10	MBAAgl7_CorAvg	T-Cell Epitope predictor	NF

Protein annotation tools listed in bold represent those not present in the other classifier type. NF: not found in the top 200 features of the other classifier type that were submitted to the greedy backward feature elimination algorithm following non-specific *F*-score filtering.

## Supplementary Materials

Supplementary materials can be found at www.mdpi.com/1422-0067/18/2/312/s1.

## Abbreviations

RV	Reverse Vaccinology
ML	Machine Learning
BPA	Bacterial Protective Antigen
BPAD	Bacterial Protective Antigen Dataset
SVM	Support Vector Machine
LTOCV	Leave Tenth Out Cross Validation
iBPAD	Intracellular Bacterial Protective Antigen Dataset
eBPAD	Extracellular Bacterial Protective Antigen Dataset
ELISA	Enzyme-linked Imunosorbent Assay
FACS	Fluorescence activated cell-sorting
DOMV	Detergent Extracted Outer Membrane Vesicle
ACC	Auto Cross Covariance
DA-PLS	Discriminant Analysis by Partial Least Squares
RBF	Radial Bias Function
TPR	True Positive Rate
FPR	False Positive Rate
ROC	Receiver Operating Characteristic
AUC	Area Under the Curve
N	Nested
B	Balanced
AF	Additional Features
HDL	High Density Lipoprotein
Th cells	T-helper cells

## References

[1] Pizza M., Scarlato V., Masignani M.M., Giuliani B., Arico M., Comanducci G.T., Jennings L., Baldi E., Bartolini B., Capecchi B. (2000). Identification of vaccine candidates against serogroup B meningococcus by whole-genome sequencing. Science.

[2] Crum-Cianflone N., Sullivan E. (2016). Meningococcal Vaccinations. Infect. Dis. Ther..

[3] Yu N.Y., Wagner J.R., Laird M.R., Melli G., Rey S., Lo R., Dao P., Sahinalp S.C., Ester M., Foster L.J. (2010). PSORTb 3.0: Improved protein subcellular localization prediction with refined localization subcategories and predictive capabilities for all prokaryotes. Bioinformatics.

[4] Corpet F., Servant F., Gouzy J., Kahn D. (2000). ProDom and ProDom-CG: Tools for protein domain analysis and whole genome comparisons. Nucleic Acids Res..

[5] Henikoff S., Henikoff J.G., Pietrokovski S. (1999). Blocks+: A non-redundant database of protein alignment blocks derived from multiple compilations. Bioinformatics.

[6] Giuliani M.M., Adu-Bobie J., Comanducci M., Arico B., Savino S., Santini L., Brunelli B., Bambini S., Biolchi A., Capecchi B. (2006). A universal vaccine for serogroup B. meningococcus. Proc. Natl. Acad. Sci. USA.

[7] Watson P.S., Turner D.P. (2016). Clinical experience with the meningococcal B vaccine, Bexsero^®^: Prospects for reducing the burden of meningococcal serogroup B disease. Vaccine.

[8] He Y., Racz R., Sayers S., Lin Y., Todd T., Hur J., Li X., Patel M., Zhao B., Chung M. (2013). Updates on the web-based VIOLIN vaccine database and analysis system. Nucleic Acids Res..

[9] Jaiswal V., Chanumolu S.K., Gupta A., Chauhan R.S., Rout C. (2013). Jenner-predict server: Prediction of protein vaccine candidates (PVCs) in bacteria based on host-pathogen interactions. BMC Bioinform..

[10] Moise L., Gutierrez A., Kibria F., Martin R., Tassone R., Liu R., Terry F., Martin B., de Groot A.S. (2015). iVAX: An integrated toolkit for the selection and optimization of antigens and the design of epitope-driven vaccines. Hum. Vaccin. Immunother..

[11] Heinson A.I., Woelk C.H., Newell M.L. (2015). The promise of reverse vaccinology. Int. Health.

[12] Sinha K., Bhatnagar R. (2010). GroEL provides protection against *Bacillus anthracis* infection in BALB/c mice. Mol. Immunol..

[13] Velikovsky C.A., Cassataro J., Giambartolomei G.H., Goldbaum F.A., Estein S., Bowden R.A., Bruno L., Fossati C.A., Spitz M. (2002). A DNA vaccine encoding lumazine synthase from Brucella abortus induces protective immunity in BALB/c mice. Infect. Immun..

[14] Fu S., Xu J., Li X., Xie Y., Qiu Y., Du X., Yu S., Bai Y., Chen Y., Wang T. (2012). Immunization of mice with recombinant protein CobB or AsnC confers protection against *Brucella abortus* infection. PLoS ONE.

[15] Jain S., Kumar S., Dohre S., Afley P., Sengupta N., Alam S.I. (2013). Identification of a protective protein from stationary-phase exoproteome of *Brucella abortus*. Pathog. Dis..

[16] Chang Y.F., Chen C.S., Palaniappan R.U., He H., McDonough S.P., Barr S.C., Yan W., Faisal S.M., Pan M.J., Chang C.F. (2007). Immunogenicity of the recombinant leptospiral putative outer membrane proteins as vaccine candidates. Vaccine.

[17] Mizrachi Nebenzahl Y., Bernstein A., Portnoi M., Shagan M., Rom S., Porgador A., Dagan R. (2007). Streptococcus pneumoniae surface-exposed glutamyl tRNA synthetase, a putative adhesin, is able to induce a partially protective immune response in mice. J. Infect. Dis..

[18] Fritzer A., Senn B.M., Minh D.B., Hanner M., Gelbmann D., Noiges B., Henics T., Schulze K., Guzman C.A., Goodacre J. (2010). Novel conserved group A streptococcal proteins identified by the antigenome technology as vaccine candidates for a non-M protein-based vaccine. Infect. Immun..

[19] Henningham A., Chiarot E., Gillen C.M., Cole J.N., Rohde M., Fulde M., Ramachandran V., Cork A.J., Hartas J., Magor G. (2012). Conserved anchorless surface proteins as group A streptococcal vaccine candidates. J. Mol. Med..

[20] Bowman B.N., McAdam P.R., Vivona S., Zhang J.X., Luong T., Belew R.K., Sahota H., Guiney D., Valafar F., Fierer J. (2011). Improving reverse vaccinology with a machine learning approach. Vaccine.

[21] Doytchinova I.A., Flower D.R. (2007). VaxiJen: A server for prediction of protective antigens, tumour antigens and subunit vaccines. BMC Bioinforma..

[22] Juncker A.S., Willenbrock H., von Heijne G., Brunak S., Nielsen H., Krogh A. (2003). Prediction of lipoprotein signal peptides in Gram-negative bacteria. Protein Sci..

[23] Larsen J.E., Lund O., Nielsen M. (2006). Improved method for predicting linear B-cell epitopes. Immunome Res..

[24] Kline K.A., Fälker S., Dahlberg S., Normark S., Henriques-Normark B. (2009). Bacterial adhesins in host-microbe interactions. Cell Host Microbe.

[25] DeLong E.R., DeLong D.M., Clarke-Pearson D.L. (1988). Comparing the areas under two or more correlated receiver operating characteristic curves: A nonparametric approach. Biometrics.

[26] Sachdeva G., Kumar K., Jain P., Ramachandran S. (2005). SPAAN: A software program for prediction of adhesins and adhesin-like proteins using neural networks. Bioinformatics.

[27] Nielsen M., Lundegaard C., Blicher T., Lamberth K., Harndahl M., Justesen S. (2007). NetMHCpan, a method for quantitative predictions of peptide binding to any HLA-A and -B locus protein of known sequence. PLoS ONE.

[28] Cai R., Liu Z., Ren J., Ma C., Gao T., Zhou Y., Yang Q., Xue Y. (2012). GPS-MBA: Computational analysis of MHC class II epitopes in type 1 diabetes. PLoS ONE.

[29] Petersen B., Petersen T.N., Andersen P., Nielsen M., Lundegaard C. (2009). A generic method for assignment of reliability scores applied to solvent accessibility predictions. BMC Struct. Biol..

[30] Nielsen M., Lundegaard C., Lund O., Kesmir C. (2005). The role of the proteasome in generating cytotoxic T-cell epitopes: Insights obtained from improved predictions of proteasomal cleavage. Immunogenetics.

[31] Liu Z., Cao J., Gao X., Ma Q., Ren J., Xue Y. (2011). GPS-CCD: A novel computational program for the prediction of calpain cleavage sites. PLoS ONE.

[32] Norata G.D., Pirillo A., Ammirati E., Catapano A.L. (2012). Emerging role of high density lipoproteins as a player in the immune system. Atherosclerosis.

[33] Norata G.D., Catapano A.L. (2012). HDL and adaptive immunity: A tale of lipid rafts. Atherosclerosis.

[34] Rudd P.M., Elliott T., Cresswell P., Wilson I.A., Dwek R.A. (2001). Glycosylation and the immune system. Science.

[35] Liu S., Cai X., Wu J., Cong Q., Chen X., Li T., Du F., Ren J., Wu Y.T., Grishin N.V. (2015). Phosphorylation of innate immune adaptor proteins MAVS, STING, and TRIF induces IRF3 activation. Science.

[36] Snapper C.M., Rosas F.R., Jin L., Wortham C., Kehry M.R., Mond J.J. (1995). Bacterial lipoproteins may substitute for cytokines in the humoral immune response to T cell-independent type II antigens. J. Immunol..

[37] Haste Andersen P., Nielsen M., Lund O. (2006). Prediction of residues in discontinuous B-cell epitopes using protein 3D structures. Protein Sci..

[38] Huang Jian H., Honda W. (2006). CED: A conformational epitope database. BMC Immunol..

[39] Ansari H.R., Raghava G.P. (2010). Identification of conformational B-cell Epitopes in an antigen from its primary sequence. Immunome Res..

[40] Fassler J.C.P. (2011). BLAST Glossary, BLAST® Help.

[41] Good P. (2013). Permutation Tests: A Practical Guide To Resampling Methods For Testing Hypotheses.

[42] Chang C.-C., Lin C.-J. (2011). LIBSVM: A library for support vector machines. ACM TIST.

[43] Chen Y.-W., Lin C.-J. (2006). Combining SVMs with Various Feature Selection Strategies. Feature Extraction.

[44] Polat K., Güneş S. (2009). A new feature selection method on classification of medical datasets: Kernel *F*-score feature selection. Expert Syst. Appl..

[45] Vergara J.R., Estévez P.A. (2014). A review of feature selection methods based on mutual information. Neural Comput. Appl..

[46] Noble W.S. (2006). What is a support vector machine?. Nat. Biotechnol..

[47] Ihaka R., Gentleman R. (1996). R: A language for data analysis and graphics. J. Comp. Graph. Stat..

[48] Simon R., Radmacher M.D., Dobbin K., McShane L.M. (2003). Pitfalls in the use of DNA microarray data for diagnostic and prognostic classification. J. Natl. Cancer Inst..

[49] Fawcett T. (2006). An introduction to ROC analysis. Pattern Recogn. Lett..

[50] Coombes K. ClassDiscovery: Classes and Methods for “Class Discovery“ with Microarrays or Proteomics, R Package Version 2.1. http://bioinformatics.mdanderson.org/Software/OOMPA.

[51] Galili T. (2015). Dendextend: An R package for visualizing, adjusting, and comparing trees of hierarchical clustering. Bioinformatics.

